# The Development of Definitive Methods for Organic Serum Constituents

**DOI:** 10.6028/jres.093.069

**Published:** 1988-06-01

**Authors:** M. J. Welch, A. Cohen, P. Ellerbe, R. Schaffer, L. T. Sniegoski, E. White

**Affiliations:** Center for Analytical Chemistry, National Bureau of Standards, Gaithersburg, MD 20899

Methods in clinical chemistry must be fast, cost-effective, and relatively easy to perform. But, of more importance, they must be sufficiently accurate and precise for proper interpretation of the results by physicians. For the most common serum analytes, a variety of methods and instrumentation are used for each. These methods vary in performance and generally exhibit a bias versus each other. An accuracy base is needed to provide a means of evaluating reference and field methods and the performance of the laboratories using them. The concept of definitive methods was developed to provide such an accuracy base. The mechanism for this accuracy transfer is through serum pools with concentrations certified by definitive methods.

For a method to be considered definitive, it must be based upon sound theoretical principles, its accuracy must be tied to an absolute physical quantity, it must be thoroughly tested for sources of bias and imprecision, and it must produce results which approximate the “true value” within narrow limits.

In cooperation with the College of American Pathologists (CAP), the National Bureau of Standards (NBS) has a long-running program on development of definitive methods. Isotope dilution mass spectrometry (IDMS) is the technique of choice for definitive methods and has been used for many serum analytes, both organic and inorganic. The organic analytes for which candidate DM’s have been developed at NBS include cholesterol [1], glucose [2], urea [3], uric acid [4], and creatinine [5], These methods have been used to certify analyte concentrations in a freeze-dried human serum Standard Reference Material as well as a number of frozen and freeze-dried serum pools supplied by the Centers for Disease Control, the College of American Pathologists, and others.

Although the individual methods vary, they have several common features, All involve addition of a weighed amount of a stable isotope labeled analogue of the compound of interest to a weighed amount of serum, followed by an equilibration period. The labeled and unlabeled forms are isolated from most of the matrix, derivatized, and subjected to GC/MS measurement of the intensity ratio of an ion from the unlabeled analyte and the corresponding ion from the labeled compound. This intensity ratio is compared with intensity ratios of standard mixtures measured immediately before and after the sample measurement. With this approach relative standard deviations for independent preparations have ranged from 0.2–0.4%. Measurements made using other pairs of ions and different gas chromatography conditions are employed to assure the absence of bias in the measurements. The labeled analogue, the derivative, and the ions monitored for each analyte are listed in table 1.

The technique has evolved with the field of analytical chemistry. The early methods developed for cholesterol and uric acid utilized packed column GC for sample introduction and magnetic switching between the two ions used for measurement. The peaks were broad enough to permit a sufficient number of measurement cycles across the peak for good precision. Support-coated open tubular columns (SCOT) were used for glucose, urea, and creatinine. Retention times were long to permit sufficient cycles. New methods for cholesterol and uric acid utilize fused-silica open tubular columns of high efficiency. To get sufficient measurement cycles across the narrow peaks required a different means of switching between ions. Switching by varying the accelerating voltage has never produced the necessary precision on the instrument used for this work. Instead, switching was accomplished by adding a DC voltage to the beam deflection plates located in front of the detector slit. This technique has proven to work very well over a small mass range (about 1%) with no loss of precision.

SRM 909, a freeze-dried human serum pool, has been analyzed by each of the definitive methods. These results are shown in table 2. Repeated measurements of glucose over several years have shown that glucose is unstable in this matrix. The decline in glucose concentration has averaged 0.7% per year. The other analytes are much more stable; cholesterol has shown a very small decrease over 6 years, uric acid is being measured this year, but does not appear to be significantly different. Urea and creatinine have not been remeasured, but the original certification measurements were done when the material was several years old and the material was found to be completely homogeneous from vial to vial with respect to these two analytes. We found for glucose and would expect to find for the other analytes that if they are degrading with time the vial to vial variations increase with time.

The new capillary column method for cholesterol has been compared to the old packed column method using SRM 909 and sera from the CAP proficiency testing program. The analyses were run about 1 year apart. The differences observed were small (<0.2%) and in both directions. The results from the new method are more precise and less likely to be biased by hidden interferences.

Future plans for the definitive method program include extending the technology to other analytes and to other matrices. The techniques developed here have been used to measure ascorbic acid in milk and cholesterol in a spiked coconut oil. For any new analytes, there must be available a high-purity well-characterized certified reference compound to tie the accuracy of the method to an absolute quantity. Planning is underway to explore the quantitative potential of isotope dilution liquid chromatography-mass spectrometry. If acceptable precision can be obtained by this technique, the range of compounds for which definitive methods are possible would be increased.

## Figures and Tables

**Table 1:** Labeled analogues, derivatives, and principal ions monitored for each analyte

Analyte	Labeled analogue	Derivative	Ions measured
Cholesterol	chatesterol_7_	TMS ether	458,465
	cholesterol-^13^C_3_	“	458,461
Glucose (2 methods)	glucose-^13^C_6_	diacetonate	245,250
	“	butylboronate-acetate	297,302
Urea	urea-^18^O	6-methyluracil-TMS	255,257
Uric Acid (2 methods)	uric acid-^15^N_2_	tetraethyl	280,282
	“	t-butyldimethylsilyl	567,569
Creatinine	creatinine-^13^C_2_	ethyl ester of N-(4,6-dimethyl-2-pyrimidinyl)-N-methylglycine	150,152

**Table 2:** Certified concentrations of organic analytes in SRM 909 lyophilized human serum with 99% confidence intervals

Analyte	Certified concentration mmol/L·g
Cholesterol	4.359 ± 0.017
Glucose	7.74 + 0.04
	−0.15
Uric acid	0.570 ± 0.003
Urea	11.387 ± 0.049
Creatinine	0.1796 ± 0.0007
